# Editorial: Genetic advancements for improving the plant tolerance to biotic and abiotic stresses

**DOI:** 10.3389/fgene.2024.1426680

**Published:** 2024-05-27

**Authors:** Krishnanand P. Kulkarni, Amaranatha R. Vennapusa, Balaji Aravindhan Pandian, Rupesh Deshmukh

**Affiliations:** ^1^ Department of Agriculture and Natural Resources, Delaware State University, Dover, DE, United States; ^2^ Enko, Mystic, CT, United States; ^3^ Department of Biotechnology, Central University of Haryana, Mahendragarh, India

**Keywords:** genetic advancement, abiotic stress, disease resistance, quantitative trait loci, genome-wide association studies, molecular mechanisms

## Introduction

Climate change is multi-faceted and primarily includes rising temperatures, increasing frequency of extreme weather events, enhanced accumulation of greenhouse gases (e.g., CO_2_, methane) in the atmosphere, and alterations in precipitation patterns (Gray and Brady, 2016; Vennapusa et al., 2023). Such events exaggerate abiotic stress factors while providing favorable situations to biotic stressors such as pests and diseases. Therefore, understanding and safeguarding the complex interplay of these stressors is imperative for developing resilient crop varieties and ensuring global food and nutritional security (Kulkarni et al., 2018). In this regard, plant scientists face a major challenge to develop strategies for enhancing crop resilience and ensuring food security.

In nature, plants are concurrently exposed to multiple abiotic stress factors (Nabi et al., 2019), which allow them to co-evolve and develop endurance through a wide range of finely balanced responses (Lima et al., 2015; Gonzalez Guzman et al., 2022). Understanding the molecular, genetic, and regulatory mechanisms involved in plant’s responses will help devise strategies to mitigate climate change. Advancements in next-generation sequencing techniques led to the development of high-quality reference genomes, high-throughput genotyping systems, and complex genetic linkage maps, which enabled the precise identification of genomic regions associated with a trait of interest through genome-wide association studies (GWAS) and quantitative trait loci (QTL) mapping (Asekova et al., 2021; Uffelmann et al., 2021). Researchers are able to significantly accelerate the pace of crop genetic improvements for simple and complex traits through marker-assisted selection (MAS) or genomic selection (GS). Owing to these developments, substantial progress has been made in understanding the mechanisms of plant tolerance to abiotic and biotic stresses and adaptation. With recent advances in gene-editing technologies, it is now feasible to develop plants with favorable traits, such as increased productivity, improved adaptability to abiotic and biotic stresses, and better nutritional composition (Wang et al., 2017; Movahedi et al., 2023).

In this Research Topic on “Genetic advancements for improving the plant tolerance to biotic and abiotic stresses”, we have collated 12 articles (10 original research articles and two review articles) that highlight the potential insights into the physiological, molecular, and genetic factors involved in imparting abiotic and biotic stress tolerance in plants. This compilation highlights many recent findings, methods, and molecular genetic resources for crop improvement programs and contributes to enhancing agricultural productivity and sustainability.

## Understanding plant resistance to biotic stresses

Plants must constantly defend themselves against attacks from various kinds of pathogens, causing destructive diseases. Five articles from this Research Topic describe the effective genetic and molecular mechanisms plants have developed to recognize and respond to infection by several pathogens. The study by Omondi et al. identified genomic regions associated with gray leaf spot (GLS) and northern corn leaf blight (NCLB) resistance in tropical maize (*Zea mays* L.). The QTLs explained 64.2% and 64.9% of the total phenotypic variance for GLS and NCLB resistance, respectively. Further, association mapping identified 11 significant single nucleotide polymorphisms (SNPs) associated with GLS and 16 SNPs with NCLB resistance; many of these SNPs found to be co-localized with QTL regions identified in a biparental population. Using the same sets of plants, the authors tested genomic prediction models and highlighted the application of GS for improving resistance to multiple diseases in maize.

For genetic dissection of plant resistance to yellow rust (YR) and leaf rust (LR), Kokhmetova et al. evaluated two recombinant inbred line populations for multiple years and identified nine and four stable QTLs for adult plant resistance to YR and LR, respectively. Further *in silico* analysis of these regions revealed candidate genes that regulate host response toward pathogen infection. The stable QTLs and genes identified in this study are helpful genetic resources for developing rust-resistant varieties through MAS.

Cotton (*Gossypium* sp.) production is continuously being challenged by various biotic and abiotic stresses, among which cotton leaf curl disease (CLCuD) is most prevalent and causes severe losses. The review by Nadeem et al. comprehensively addresses the challenges faced by CLCuD in cotton crop cultivation, strategies for disease management, and potential options to improve the resistance. The review highlights the basis of CLCuV strains and genetic architecture, transmission vectors and mechanisms, and the potential of advanced biotechnological and molecular breeding approaches, such as genetic engineering, speed breeding, and genome editing, in developing CLCuD-resistant cotton varieties. Overall, the review emphasizes adopting these advancements to revolutionize crop improvement and ensure sustainable cotton production.

The pioneering study by Ellur et al. provides the first comprehensive characterization of specific chickpea (*Cicer arietinum*) Polygalacturonase-inhibiting proteins (PGIPs) using computational, localization, and gene expression studies. Identifying CaPGIP3 and CaPGIP4 on chromosome 3 in this study expands the genomic understanding of CaPGIPs, dictating a reconsideration of their genomic organization from previous reports on chromosome 6. The findings highlight the potential of *CaPGIP1, CaPGIP3*, and *CaPGIP4* genes in combating chickpea pathogens, specifying their structural and functional similarities with other legume PGIPs.

Song et al. investigated the role of microRNAs (miRNAs) in combating Potato virus Y (PVY) disease, a major threat to crop quality and productivity. The comparative study of small RNA and transcriptome sequencing on healthy and PVY-infected *Nicotiana benthamiana* tissues revealed the differentially expressed miRNAs and predicted their potential gene targets. This inclusive analysis enhances our understanding of a complex regulatory network involved in the host’s response to PVY infection and connection with miRNAs, offering valuable insights into novel strategies for PVY control.

## Understanding plant tolerance to abiotic stresses

Recent advancements in understanding plants’ adaptive responses to abiotic stress have opened new avenues for improving stress tolerance and productivity under adverse environmental conditions. The articles related to abiotic stress in the current Research Topic provide an overview of key developments driving progress in crucial areas of plant science. The review by Misra et al. highlights the potential use of CRISPR/Cas 9 technology for abiotic and biotic stress management in sugar beet (*Beta vulgaris*). Candidate - means potential candidate genes, known for their role in alkaline, cold, and heavy metal stresses, can be precisely modified via CRISPR/Cas 9 technology to enhance sugar beet’s resilience to abiotic stresses with minimal off-target effects. The authors present a list of potential genes that can be targeted by a CRISPR tool for genetic improvements for heat, drought, heavy metal, and other abiotic stresses, as well as biotic stress factors such as cyst nematode (*Heterodera schachtii schmidt*) resistance, necrotic yellow vein virus resistance, and insect-pest resistance.

Salt stress affects many plant life stages, from seed germination to flowering and fruiting. Sun et al. studied salt stress influencing yield and quality in radish (*Raphanus sativus* L.). They specifically targeted the role of long non-coding RNAs (lncRNAs) in regulating salt stress response using the genome-wide identification study. Notably, this is the first comprehensive analysis of salt stress-responsive lncRNAs in radish and provides potential insights into the regulatory networks governing salt stress tolerance and their potential target sites in radish. These findings lay a foundation for understanding stress responses and future functional characterization of lncRNAs associated with radish defense response against high salinity. Transcription factors also play pivotal regulatory and molecular switch roles by controlling the expression of downstream genes or directly acting to protect plants from the damage induced by salt stress. Chen et al. analyzed the role of WRKY genes in *Tamarix ramosissima* in response to NaCl stress. Many *WRKY* genes actively upregulated their expression levels to resist NaCl stress through the plant-pathogen interaction and the MAPK signaling pathways. The authors reported that WRKY transcription factors can modulate the activation or inhibition of related genes during NaCl stress, which may enhance plant's adaptability to saline environments and mitigate NaCl-induced damage.

Brassinosteroid is a phytohormone reported to play an essential role in acclimation to environmental stresses, resistance to pathogens, and cell elongation, resulting in increased crop yield and plant growth (Kim and Russinova, 2020). Lei et al. characterized brassinosteroid signaling kinase (BSK) family genes that respond to salinity stress in cotton to decipher the functions of *BSK* genes on growth and responses to abiotic stresses and learn the evolutionary relationship of cotton BSKs. The study showed the induced expression of *BSK* genes under salt stress, suggesting the important role of *BSK* gene regulation in cotton growth, development, and stress response.

Inadequate soil fertility (particularly low nitrogen) is a primary cause of low maize yields in sub-Saharan Africa, leading to food insecurity, malnutrition, and rural poverty in smallholder farming communities. Kimutai et al. discovered QTLs associated with grain yield and agronomic traits in maize (*Zea. mays*. L) under optimum and low nitrogen conditions to address this issue. These QTLs are of significant value for further validation and possible rapid introgression into maize populations using MAS. The authors further implied that using GS can improve genetic gain in maize breeding for low nitrogen stress tolerance.

The research by Malik et al. elucidates the genes involved in flavonoid biosynthesis pathways through *de novo* transcriptome sequencing of *A. racemosus,* commonly called Shatavari. The authors identified several differentially expressed genes, including the NAC family of plant transcription factors known for their role in abiotic stress tolerance in plants. The study provides a molecular understanding of the genes involved in the flavonoid biosynthesis pathway for further secondary metabolic improvement in the *Asparagus racemosus*.

The study by Wang et al. investigated the impact of cadmium (Cd) stress on clove basil (*Ocimum gratissimum* L.) physiological traits and integrated it with transcriptome analysis to explore the molecular-level responses. The findings provide novel candidate genes and genetic resources for understanding Cd stress adaptation mechanisms and offer insights into potential strategies for crop breeding programs to improve heavy metal (HM) tolerance. This research highlights the importance of integrating the physiognomic studies with genomics in aromatic plants like clove basil, which play a significant role in safeguarding HM pollution while enhancing aromatic essential oil production and thus advancing phytoremediation efforts and sustainable utilization of HM-contaminated soils.

## Conclusion

The contributions to this Research Topic provide a catalog of physiological, molecular, and genetic information related to plants’ tolerance to abiotic and biotic stresses. The identified stress adaptive mechanisms, potential QTLs, candidate genes, and state-of-the-art approaches mentioned in these articles broaden our knowledge and provide genetic resources and avenues for crop improvement programs toward enhanced productivity and climate resilience. This Research Topic addressed vital factors and advancements that will accelerate genetic improvements in producing climate-resilient plants.
